# Supplementary material for: Comparing the Synergistic and Antagonistic Interactions of Ciprofloxacin and Levofloxacin Combined with Rifampin against Drug-Resistant Staphylococcus aureus: A Time–Kill Assay
Source: Antibiotics (Basel). 2023 Apr 6;12(4):711. doi: 10.3390/antibiotics12040711 (PMC10135007; doi:10.3390/antibiotics12040711)

Supplementary Materials

Table S1 The median and interquartile range of viable bacterial counts after exposure to antibacterial agents (1× MIC) for a specific time period

	Median (interquartile range) log CFU/ml			
	0h	4h	8h	24h
Control	6.135 (5.915 – 6.298)	7.615 (6.312 – 8.527)	8.695 (5.930 – 9.255)	9.665 (5.830 – 10.217)
Rifampin	6.135 (6.000 – 6.300)	6.530 (4.935 – 7.160)	5.850 (3.792 – 7.178)	6.535 (4.527 – 8.992)
Ciprofloxacin	6.130 (5.938 – 6.300)	6.800 (6.485 – 7.190)	6.700 (5.340 – 7.265)	6.975 (5.860 – 8.205)
Levofloxacin	6.115 (5.945 – 6.287)	4.320 (3.540 – 5.875)	3.090** (1.560 – 3.510)	5.835 (4.060 – 6.820)
Rifampin + Ciprofloxacin	6.050 (5.992 – 6.255)	5.850 (4.482 – 6.348)	5.830 (3.985 – 6.155)	3.385* (0.3525 – 3.955)
Rifampin + Levofloxacin	6.110 (5.982 – 6.282)	6.135 (4.620 – 6.327)	4.600 (3.980 – 5.855)	3.660* (3.143 – 4.235)

* Synergy

** Bactericidal

Table S2 The median and interquartile range of viable bacterial counts after exposure to antibacterial agents (0.5× MIC) for a specific time period

	Median (interquartile range) log CFU/ml			
	0h	4h	8h	24h
Control	6.150 (5.945 – 6.320)	8.595 (8.390 – 8.870)	9.425 (9.245 – 9.785)	10.210 (10.110 – 10.260)
Rifampin	6.135 (5.952 – 6.325)	6.010 (5.537 – 6.550)	6.005 (4.353 – 6.282)	7.525 (4.650 – 9.447)
Ciprofloxacin	6.160 (5.985 – 6.320)	7.045 (6.820 – 7.312)	7.285 (6.770 – 7.630)	9.265 (7.465 – 9.960)
Levofloxacin	6.100 (5.965 – 6.287)	6.235 (4.803 – 7.565)	5.370 (3.720 -7.735)	9.385 (7.383 – 10.137)
Rifampin + Ciprofloxacin	6.120 (5.963 – 6.277)	6.310 (5.840 – 6.537)	5.830 (4.220 – 6.185)	3.970* (3.502 – 6.055)
Rifampin + Levofloxacin	6.085 (5.960 – 6.260)	6.030 (4.872 – 6.287)	5.870 (4.495 – 6.317)	4.415 (3.627 – 6.130)

* Synergy

Figure S1 Antibacterial activity of rifampin combined with ciprofloxacin or levofloxacin against drug-resistant *Staphylococcus aureus* strains (mean CFU/mL with standard deviation). MIC, minimum inhibitory concentration; RIF, rifampin; CIP, ciprofloxacin; LVX, levofloxacin

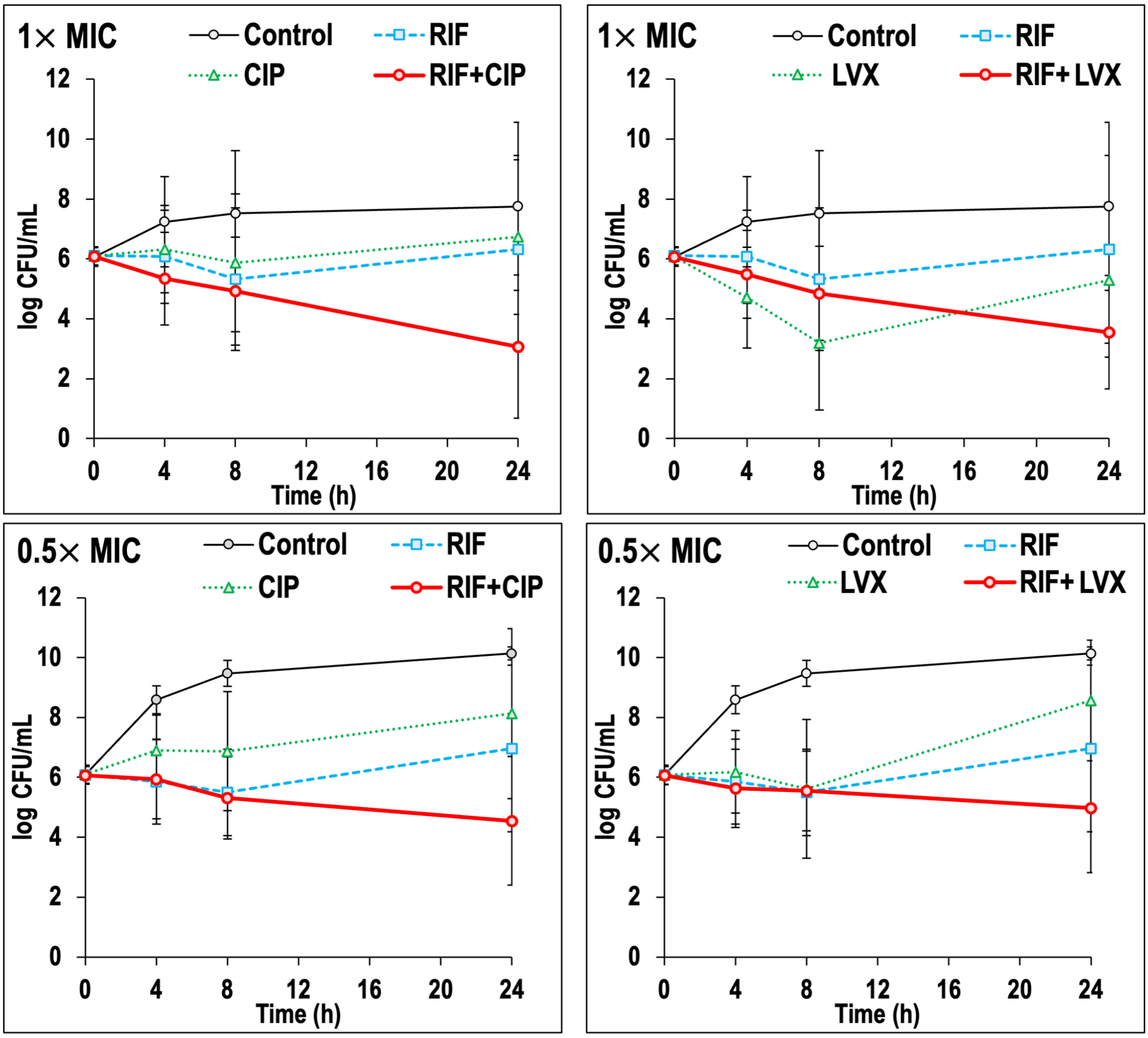

Supplement: Supplementary file 1 [file antibiotics-12-00711-s001.zip › antibiotics-2290703-supplementary.pdf]
